# Clinical manifestations, treatment options, and comorbidities in COVID‐19 relapse patients: A systematic review

**DOI:** 10.1002/jcla.24402

**Published:** 2022-04-08

**Authors:** Maryam Koupaei, Mohamad Hosein Mohamadi, Ilya Yashmi, Amir Hossein Shahabi, Amir Hosein Shabani, Mohsen Heidary, Saeed Khoshnood

**Affiliations:** ^1^ 48462 Department of Microbiology and Immunology School of Medicine Kashan University of Medical Sciences Kashan Iran; ^2^ 56941 Student Research Committee Sabzevar University of Medical Sciences Sabzevar Iran; ^3^ 56941 Department of Laboratory Sciences School of Paramedical Sciences Sabzevar University of Medical Sciences Sabzevar Iran; ^4^ 56941 Cellular and Molecular Research Center Sabzevar University of Medical Sciences Sabzevar Iran; ^5^ Clinical Microbiology Research Center Ilam University of Medical Sciences Ilam Iran

**Keywords:** COVID‐19, recurrence, reinfection, relapse, SARS‐CoV2

## Abstract

**Introduction:**

Interest revolving around coronavirus disease 2019 (COVID‐19) reinfection is escalating rapidly. By definition, reinfection denotes severe acute respiratory syndrome coronavirus 2 (SARS‐CoV‐2), PCR redetection, and COVID‐19 recurrence within three months of the initial symptoms. The main aim of the current systematic review was to evaluate the features of COVID‐19 relapse patients.

**Materials and methods:**

For this study, we used a string of terms developed by a skilled librarian and through a systematical search in PubMed, Web of Science, and Embase for eligible studies. Clinical surveys of any type were included from January 2019 to March 2021. Eligible studies consisted of two positive assessments separated by a negative result via RT‐PCR.

**Results:**

Fifty‐four studies included 207 cases of COVID‐19 reinfection. Children were less likely to have COVID‐19 relapse. However, the most patients were in the age group of 20–40 years. Asthenia (66.6%), headache (66.6%), and cough (54.7%) were prevalent symptoms in the first SARS‐CoV‐2 infection. Asthenia (62.9%), myalgia (62.9%), and headache (61.1%) were most frequent in the second one. The most common treatment options used in first COVID‐19 infection were lopinavir/ritonavir (80%), oxygen support (69.2%), and oseltamivir (66.6). However, for the treatment of second infection, mostly antibiotics (100%), dexamethasone (100%), and remdesivir (80%) were used. In addition, obesity (32.5%), kidney failure (30.7%), and hypertension (30.1%) were the most common comorbidities. Unfortunately, approximately 4.5% of patients died.

**Conclusion:**

We found the potency of COVID‐19 recurrence as an outstanding issue. This feature should be regarded in the COVID‐19 management. Furthermore, the first and second COVID‐19 are similar in clinical features. For clinically practical comparison of the symptoms severity between two epochs of infection, uniform data of both are required. We suggest that future studies undertake a homogenous approach to establish the clinical patterns of the reinfection phenomena.

## Graphical abstract

Totally, 1807 studies obtained from PubMed/Medline, Web of Science, and Embase databases. Following the removal of duplicates, the title and abstract of 998 studies were screened to select the studies which report the relapse of COVID‐19 after a negative RT‐PCR test. Finally, among the 152 full‐text articles, 54 studies were found to be eligible for data extraction.

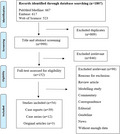

## 1 INTRODUCTION

This is not the first time that coronavirus has caused problems in the world. Viruses such as severe acute respiratory syndrome coronavirus (SARS‐CoV) and Middle East respiratory syndrome coronavirus (MERS‐CoV) have also been prevalent in recent years.^1^ The mortality rates of SARS‐CoV and MERS‐CoV epidemics have been estimated to be 10% and 35%, respectively.^2^ The main problem of today's global health communities is conflict over the novel coronavirus (2019‐nCoV). The new virus was first identified in China, but is now found in many countries around the world.^1^ There are currently several variants of coronavirus circulating among people,^3^, ^4^ and the virus is mostly transmitted through the respiratory tract. Various symptoms have been described for patients with COVID‐19, ranging from asymptomatic to severe. Kidney damage has also been reported in some cases.^5^ According to the World Health Organization (WHO), COVID‐19‐infected patients can leave home quarantine after the improvement of their infectious symptoms and also the confirmation of two negative RT‐PCR tests (within 24 h).^6^

Reinfection, relapse, recurrence, and reactivation are terms used for people infected with coronavirus and have become positive again after a period of negativity.^7^ Based on a report from Guangdong Province in China, about 14% of patients who recover from COVID‐19 become reinfected with the virus.^8^ In addition, there have been reports of reinfection in Korea and Japan.^9^ Duration of immunization against coronavirus reinfection in recovering individuals is six months.^10^ People who become infected with coronavirus for the second time often have milder symptoms and recover more quickly than those infected for the first time.^11^ However, there are concerns about reinfection in people recovering from the coronavirus. The objective of this systematic review was to evaluate the prevalence and frequency of reinfection in people recovering from COVID‐19 and their clinical signs, as well as to assess the treatment methods.

## 2 MATERIALS AND METHODS

The present systematic review was conducted in accordance with Preferred Reporting Items for Systematic Reviews and Meta‐Analyses (PRISMA) statements.^12^

### 2.1 Search strategy

We used PubMed/Medline, Web of Science, and Embase for a systematic search from January 1, 2019, to March 7, 2021. The search was based on the following string of terms: (“recurrence” OR “relapse” OR “reinfection” OR “reactivation”) AND (“COVID‐19” OR “severe acute respiratory syndrome coronavirus 2” OR “novel coronavirus” OR “SARS‐CoV‐2” OR “nCoV disease” OR “SARS2” OR “2019‐nCoV” OR “coronavirus disease‐19” OR “coronavirus disease 2019” OR “2019 novel coronavirus” OR “Wuhan coronavirus” OR “Wuhan seafood market pneumonia virus” OR “Wuhan pneumonia”). There was no limitation on language, location, and type of studies.

### 2.2 Inclusion and exclusion criteria

All the studies reported the reactivation or second infection of COVID‐19 were considered in the search. Total records were retrieved and entered into EndNote X9 software (Thomson Reuters). Following duplicate exclusion, a three‐stage screening was carried out to exploit the eligible studies based on title, abstract, and full text. The whole eligible studies reported the patients who were recovered from primary infection, but then developed a secondary COVID‐19 infection. RT‐PCR test was necessary inclusion criteria. Patients with a positive RT‐PCR for the first phase of COVID‐19, a negative RT‐PCR for recovery, and a second positive RT‐PCR for COVID‐19 recurrence were examined in the study. We excluded articles that reported only a serologic diagnosis test, without a nasopharyngeal swab RT‐PCR test, as well as duplicate publication of same studies, congress abstracts, reviews, systematic reviews and meta‐analysis, cellular and molecular studies, and animal studies. All types of manifestations and treatments were regarded without any restriction, and there was no limitation on comorbidities and underlying disorders.

### 2.3 Data extraction

The following data were acquired from each article: first author's name, location, publication time, type of study, number of relapsed patients, age, gender, interval between two infections, clinical manifestations, treatment, relative status, comorbidities, and outcome. Two investigators independently extracted the data from full text of 54 included studies. Inconsistencies between reviewers were resolved by consensus. The retrieved data are represented in Table 1.

**TABLE 1:** Characteristics of the included studies

First author	Country	Published time	Type of study	*N*. of relapsed patients	Median age at first infection	Male/female	Time between infections (days)	Clinical manifestations in first infection	Treatment in first infection	Clinical manifestations in second infection	Treatment in second infection	Status	Comorbidities	Outcomes
Lancman et al.^35^	USA	October 2020	Case report	1	55	F	42	Fever, abdominal pain, cough, nausea, vomiting	HCQ, AZ, RDV	Fever, sore throat, abdominal pain, diarrhea, respiratory distress	Plasma, DEX, oxygen support	Worse	B cell ALL, diabetes mellitus, heart failure, asthma	Discharged
Chen et al.^36^	China	July 2020	Case series	4	32	M 2 F 2	12	Fever 2, cough 3, fatigue 1	ARB 1, IFN 1, CTM 1	NR	NR	NR	NR	Discharged 4
Nazir et al.^37^	India	October 2020	Case report	1	26	M	99	None	HCQ, OTV, montair, RAN, vit B, vit C, zinc	None	HCQ, OTV, montair, RAN, vit B, vit C, zinc	ND	NR	Discharged
Selhorst et al.^38^	Belgium	November 2020	Case report	1	39	F	185	Cough, dyspnea, headache, fever, malaise	NR	Dyspnea, rhinitis, sore throat	NR	Better	None	Discharged
Selvaraj et al.^33^	USA	December 2020	Case report	1	70–80	M	240	Respiratory distress	ALB, Antitussives	Respiratory distress, fever, Myalgia, nausea, malaise	AZ, DEX, RDV, CRO, oxygen support	Worse	Obesity, neuropathy, asthma, sleep apnea, hypertension	Discharged
Bellesso et al.^39^	Brazil	December 2020	Case report	1	76	F	126	Respiratory distress	CRO, VAN	Respiratory distress, hypoxemia, dyspnea	Meropenem, VAN, DEX, polymixin, linezolid, oxygen support	Worse	Hypercalcemia, anemia, Kidney failure, hypertension, under hemodialysis, MM, plasmacytoma, glucose intolerance	Died
Zhou et al.^40^	China	July 2020	Case report	1	40	M	5	Fever, dyspnea, diarrhea	Oxygen support, ARB, mPDRL, Ig	Fever	Oxygen support, mPDRL	Better	Pneumonia	Discharged
Atici et al.^41^	Turkey	January 2021	Case series	2	46.5	M 1 F 1	102	Fever 1, sore throat 1, headache 2, cough 1, asthenia 1, nausea 1, diarrhea 1, abdominal pain 1, myalgia 1	HCQ 2, AZ 1, CRO 1	Sore throat 2, fever 2, headache 2, myalgia 2, asthenia 1, nausea 1, cough 1, respiratory distress 1	Favipiravir 2, AZ 1, CRO 1	ND	None	Discharged 2
Shoar et al.^42^	Iran	February 2021	Case report	1	31	M	79	Fever, malaise, cough, respiratory distress, anosmia	Oxygen support, HCQ, DEX	Malaise, gingival aphthous ulcers, painful submandibular lymphadenopathy, fever, myalgia, skin desquamation during recovery	Naproxen	NR	NR	Discharged
Mulder et al.^43^	Netherland	October 2020	Case report	1	89	F	59	Fever, fatigue, cough	NR	Fever, cough, dyspnea	NR	Worse	Waldenstrom macroglobulinemia	Died
Hanif et al.^44^	Pakistan	October 2020	Case report	1	58	M	49	Fatigue, headache, sore throat	Oxygen support, AZ	Fever, headache, myalgia	NR	Better	Pneumonia	Discharged
Abdallah et al.^45^	USA	December 2020	Case report	1	30	M	30	Chest pain, fever, and night sweat, progressive fatigue, anosmia	APAP	Chest pain, fatigue, dyspnea	AZ	ND	None	Discharged
Brito et al.^46^	Brazil	October 2020	Case series	2	42	M 1 F 1	21	Fever 1, cough 2, sore throat 2, myalgia 2, fatigue 2, diarrhea 2, headache 2	AZ 1, IVR 1	Fever 2, cough 2, sore throat 2, myalgia 2, fatigue 2, diarrhea 2, headache 2, anosmia 2, dysgeusia 2, asthenia 1, nausea 1	HCQ 2, AZ 2, IVR 2	NR	None	Discharged 2
Hussein et al.^47^	Iraq	December 2020	Case report	1	46	M	53	Fever, cough	AZ, vit D, zinc	Fever, sore throat, cough, ageusia, anosmia	Favipiravir	NR	NR	Discharged
Kapoor et al.^48^	India	February 2021	Case series	3	33	M 3	78.3	Fever 1, cough 1	RDV 1, oxygen support 1	Fever 3, chills 1, respiratory distress 1, headache 1, vomiting 1	RDV 1, plasma 1, IVIg 1, oxygen support 3	Worse 2 Better 1	MM 1, ALL 2, Pneumonia 1	NR
Liu et al.^49^	China	August 2020	Case report	1	57	F	5	Fever, cough	LPV, IFN, ARB hydrochloride	NR	LPV, IFN, ribavirin, budesonide	NR	NR	Discharged
Salcin et al.^50^	USA	December 2020	Case report	1	62	F	120	Cough, respiratory distress	Antibiotics, HCQ, vit C, zinc	Tachycardia, tachypnea, hypoxia	Oxygen support, DEX, RDV, CRO, AZ, vit C, zinc, plasma, antibiotics, steroid	Worse	Hypertension, hypothyroidism, degenerative disk disease, previous L2‐L4 lumbar fusion, anxiety	Discharged
Santos et al.^51^	Brazil	February 2021	Case control	33	39.2	M 7 F 26	50.5	Headache 29, asthenia 27, myalgia 16, arthralgia 10, sneeze 15, sore throat 19, dysgeusia 10, dyspnea 11, diarrhea 16, hyporexia 12, abdominal pain 10, nausea 10, vomiting 10, fever 7, cough 15, anosmia 8, skin lesions 8, dizziness 9, mental confusion 2	AZ 20, corticosteroid 12, IVR 9, heparin 5, HCQ 5	Headache 28, asthenia 29, myalgia 24, arthralgia 14, sneeze 22, sore throat 20, dysgeusia 17, dyspnea 19, diarrhea 16, hyporexia 15, abdominal pain 12, nausea 12, vomiting 12, fever 12, cough 21, anosmia 16, skin lesions 5, dizziness 12, mental confusion 5	AZ 20, corticosteroid 26, IVR 21, heparin 12, HCQ 3, antibiotics 20, oxygen support 3	NR	Obesity 10, diabetes mellitus 1, hypertension 5, asthma 1	Died 1 Discharged 32
Goldman et al.^52^	USA	September 2020	Case report	1	60–69	NR	100	Fever, chills, cough, dyspnea, chest pain	Oxygen support, steroids	Cough, asthenia, dyspnea	Oxygen support, RDV, DEX	Better	Emphysema, hypertension, pneumonia	NR
Duggan et al.^53^	USA	June 2020	Case report	1	82	M	10	Fever, tachypnea, hypoxia, respiratory distress	Oxygen support	Fever, hypoxia, tachycardia, tachypnea, hypotension	Oxygen support	Worse	Parkinson's disease, diabetes, kidney failure, hypertension	Discharged
Coppola et al.^54^	Italy	August 2020	Case report	1	68	M	16	Diarrhea, asthenia, fever, dyspnea, cough, myalgia	Tocilizumab, LPV/r, HCQ, oxygen support	Diarrhea, fever, myalgia, asthenia	NR	Better	Smoking, dyslipidemia, heart failure, carbohydrate intolerance	Discharged
Sicsic Jr et al.^55^	USA	February 2021	Case report	1	69	F	72	Respiratory distress, cough, headache, fatigue, fever	AZ, OTV	Cough, fever, ageusia	RDV, antibiotics, DEX, oxygen support	Worse	Asthma, hypercholesterolemia, hypertension, sleep apnea	Discharged
Dou et al.^56^	China	July 2020	Case report	1	34	M	18	Fever, chills, cough, sore throat, dizziness, fatigue	ARB, ribavirin, Ig, cefuroxime, LPV/r, IFN, cefoperazone sodium, sulbactam sodium, CTM	None	ARB, CQ phosphate, IFN	Better	Diabetes	Discharged
Novoa et al.^57^	Colombia	January 2021	Case report	1	44	M	89	None	NR	Malaise, chills, headache, fever, sore throat	NR	Worse	None	Discharged
Tuan et al.^58^	USA	February 2021	Case report	1	43	M	7	Respiratory distress, hypoxia, fever, myalgia, sore throat	Tocilizumab, HCQ, Ig, mPDRL, oxygen support	Respiratory distress	VAN, TZP, RDV, oxygen support	Worse	Diabetes, obesity, hypothyroidism	Discharged
Sharma et al.^59^	Qatar	December 2020	Case report	1	57	M	85	None	CQ/HCQ, OTV	Fever, myalgia, headache, cough	AZ, OTV	Worse	Diabetes	Discharged
Bonifácio et al.^60^	Brazil	September 2020	Case report	1	24	F	36	Headache, malaise, asthenia, fever, sore throat, nasal congestion	Naproxen, dipyrone	Malaise, myalgia, severe headache, fatigue, asthenia, fever, sore throat, anosmia, dysgeusia, diarrhea, cough, hyposmia	NR	Worse	Obesity	Discharged
Scaria et al.^61^	India	September 2020	Case series	2	26.5	M 1 F 1	100.5	None	NR	None	NR	Worse 2	NR	Discharged 2
Ma et al.^62^	Hong Kong	September 2020	Case report	1	31	F	2	Fever, dyspnea	NR	Myalgia, cough, fever	LPV/r	NR	Kidney failure, pneumocystis pneumonia, hypertension	Discharged
Zhang et al.^63^	China	Nov ember 2020	Case series	4	48.75	M 1 F 3	15.5	Fever 4, cough 3, respiratory distress 1, sore throat 1, headache 1, myalgia 1	LPV/r 4, HCQ 2, IFN 4, thymalfasin 1, CTM 4, ARB 2, thymopentin 1	None	Thymalfasin 3, HCQ 4, CTM 4, IFN 4, ARB 1	Better 4	Hepatitis B	Discharged 4
Fehdi et al.^64^	Morocco	May 2020	Case report	1	69	M	3	Fever, cough, dyspnea, respiratory alkalosis, hypoxemia	HCQ, AZ, CRO, moxifloxacin, thromboprophylaxis	Respiratory distress	Oxygen support	Worse	Inflammatory syndrome	Died
Caralis^65^	USA	November 2020	Case series	7	60	M 5 F 2	NR	Cough 3, fever 4, fatigue 1, dyspnea 1, diarrhea 1, ageusia 1, anosmia 1, headache 1	NR	Fever 1, headache 1, anosmia 1, ageusia 1, fatigue 2	NR	Better 4, ND 3	Arthritis 3, kidney failure 3, liver failure 3, HIV 3, sarcoidosis 3, diabetes 1, pneumonia 1	Discharged 7
Harrington et al.^66^	UK	2021	Case report	1	78	M	223	Fever	None	Respiratory distress, aphasia, hypoxia	Co‐amoxiclav, clarithromycin, DEX	Worse	Diabetes, diabetic nephropathy disease, COPD, sleep apnea, heart failure	NR
Ozaras et al.^67^	Turkey	October 2020	Case report	1	23	F	99	Fever, chills, fatigue, cough, headache, sore throat, myalgia	APAP, AZ, HCQ	Fever, chills, fatigue, anorexia, ageusia, anosmia, myalgia	HCQ, APAP	NR	Smoking	Discharged
Prado‐Vivar et al.^68^	Ecuador	November 2020	Case report	1	46	M	47	Headache, drowsiness	NR	Sore throat, nasal congestion, fever, back pain, cough, dyspnea	NR	Worse	NR	Discharged
Bellanti et al.^69^	Italy	October 2020	Case report	1	91	F	2	Respiratory distress	DEX, TZP, daptomycin, enoxaparin, furosemide, amiodarone, bisoprolol, oxygen support	Fever, dyspnea, tachypnea, stranguria, respiratory distress	Paracetamol, cefepime, clarithromycin, oxygen support, caspofungin, furosemide, mPDRL	Worse	Diabetes, hypertension, atrial fibrillation, kidney failure, anxiety depressive disorder, UTI	Died
Yang et al.^70^	China	November 2020	Cohort study	93	34	M 36 F 57	8	NR	Steroids 14 (mPDRL and/or DEX)	Cough 18, respiratory distress 3, fever 1	NR	NR	NR	Discharged 93
Du et al.^71^	China	August 2020	Case series	3	66	M 1 F 2	14	Fever 2, dry cough 2, chest pain 1, diarrhea 1, respiratory distress 1, headache 1	Antiviral 3, CTM 3, antibiotics 2	None	CTM 3	Better 3	Hypertension 1, diabetes 1, COPD 1, digestive disease 1, renal impairment 1	Discharged 3
Tillett et al.^72^	USA	October 2020	Case report	1	25	M	10	Sore throat, cough, headache, nausea, diarrhea	None	Fever, headache, dizziness, cough, nausea, diarrhea, hypoxia, respiratory distress, myalgia	Oxygen support	Worse	None	Discharge
Nonaka et al.^73^	Brazil	May 2021	Case report	1	45	F	142	Diarrhea, myalgia, asthenia, sore throat	PRED	Headache, malaise, diarrhea, cough, sore throat, myalgia, ageusia, myalgia, insomnia, dyspnea, respiratory distress	NR	Worse	None	Discharged
Ravioli et al.^74^	Switzerland	May 2020	Case series	2	79	F 2	20	Fever 2, cough 2	NR	Dyspnea 1, fever 1, confusion 1, cough 1	HCQ 1, AZ 1, oxygen support 1	Worse 1, ND 1	Diabetes 1, heart failure 1, stroke 1	Died 1, Discharged 1
Jesus et al.^75^	Portugal	October 2020	Case report	1	NR	M	13	None	PDRL	Fever, headache, myalgia, cough dyspnea, chest pain, tachypnea, respiratory distress	mPDRL, TZP, RDV	Worse	Pneumonia, cardiopulmonary arrest	Discharged
Zayet et al.^76^	France	February 2021	Case series	3	43	F 3	NR	Myalgia 2, fatigue 2, sore throat 1, cough 1, anosmia 1, dysgeusia 1, diarrhea 1, fever 1, chills 1	NR	Myalgia 1, fatigue 1, dyspnea 2, chills 1, headache 2, cough 2, chest pain 1, anosmia 2, dysgeusia 2, vomiting 1, diarrhea 1	NR	ND	Asthma 1	NR
Ak et al.^77^	Turkey	December 2020	Case report	1	40	M	80	Fever, cough	HCQ	Sore throat, cough, diarrhea, fever	HCQ, enoxaparin, moxifloxacin	Worse	None	Discharged
Chen et al.^78^	China	March 2020	Case report	1	46	F	3	Fever, sore throat, cough, respiratory distress	OTV, ARB, LPV/r, moxifloxacin	NR	NR	Better	NR	Discharged
Wu et al.^79^	China	November 2020	Case series	2	27	M 1 F 1	14	Fever 2	IFN 2, LPV 2, silybin 1, CTM 1	None	IFN	Better 2	None	Discharged 2
Lafaie et al.^80^	France	July 2020	Case series	3	86	F 3	11	Cough 2, fever 3, respiratory signs 2 asthenia 1, ageusia 1, tachypnea 1	Levofloxacin 1, ofloxacin 1, mPDRL 1, CRO 1, anticoagulation 1, PDRL 1, rovamycine 1, corticosteroids 1	Hyperthermia 1, respiratory distress 1, dehydration 1, hypernatremia 1, melena 1, dry cough 1, fever 1	Levofloxacin 1, aztreonam 1, mPDRL 2, tocilizumab 1, furosemide 1, CRO 1, cotrimoxazole 1, plasma 1, oxygen support 1	Worse 3	Hypertension 3, beta‐lactam allergy, heart failure, diabetes, kidney failure, respiratory failure, hypothyroidism, Alzheimer's disease, arterial and rheumatoid arthritis	Died 3
Mardani et al.^81^	Iran	July 2020	Case report	1	64	F	21	Dyspnea, asthenia	CRO, clindamycin, LPV/r, HCQ	Respiratory distress	Meropenem, VAN, ampicillin, acyclovir, steroids, colistin	NR	Hypertension, heart failure, metastatic colorectal cancer, chemotherapy, bacterial meningitis, pneumonia	NR
Yadav et al.^82^	India	October 2020	Case report	1	3	M	42	None	NR	None	NR	NR	Neuroblastoma, chemotherapy	Discharged
Mahallawi^83^	Saudi Arabia	September 2020	Case report	1	31	M	NR	Myalgia, fever, headache, hyporexia, anosmia, ageusia	Paracetamol	NR	NR	NR	None	Discharged
West et al.^11^	UK	December 2020	Case report	1	25	M	NR	Fever, headache, fatigue	NR	Fatigue, coryzal symptoms	NR	Better	None	Discharged
Varella et al.^84^	Brazil	August 2020	Case report	1	26	M	32	Headache, asthenia	Home care	Fever, cough, headache, myalgia, arthralgia, anosmia, fatigue	AZ, analgesics and antipyretics	Worse	NR	Discharged
Mendoza et al.^85^	USA	August 2020	Case report	1	51	M	NR	None	NR	Fever, severe dyspnea, severe respiratory distress	DEX, RDV, oxygen support	Worse	Hypertension and ESRD due to acute tubular necrosis, undergoing chronic hemodialysis thrice weekly	Discharged
Lee et al.^86^	South Korea	November 2020	Major article	6	29.5	M 2 F 4	12	Fever 2, cough 1, sore throat 1, sputum 1, rhinorrhea 2, anosmia 2, chest pain 1, diarrhea 1, fatigue 1, anorexia 1	Symptomatic care with oral antitussives and esomeprazole 1	Fever 2, cough 1, sputum 1, chest pain 1, chill 1, dyspnea 1, rhinorrhea 1	Symptomatic care 1	ND 1 Better 3 NR 2	Allergic rhinitis 1, dyslipidemia 1, Parkinson's disease 1, dementia 1, depression 1	Discharged 6

Abbreviations: ALB, albuterol; APAP, acetaminophen; ARB, arbidol; AZ, azithromycin; COPD, chronic obstructive pulmonary disease; CQ, chloroquine; CRO, ceftriaxone; CTM, Chinese traditional medicine; DEX, dexamethasone; HCQ, hydroxychloroquine; IFN, interferon; Ig, immunoglobulin; IVIG, intravenous immunoglobulin; IVR, ivermectin; LPV, lopinavir; LPV/r, lopinavir/ritonavir; MM, multiple myeloma; mPDRL, methylprednisolone; ND, no difference; NR, not reported; OTV, oseltamivir; PDRL, prednisolone; PRED, prednisone; RAN, ranitidine; RDV, remdesivir; RPV, ritonavir; TZP, piperacillin/tazobactam; UTI, urinary tract infection; VAN, vancomycin.

### 2.4 Quality assessment

The critical appraisal checklist provided by the Joanna Briggs Institute (JBI) was used to perform a quality assessment of the studies.^13^

## 3 RESULTS

### 3.1 Study characteristics

**FIGURE 1:**
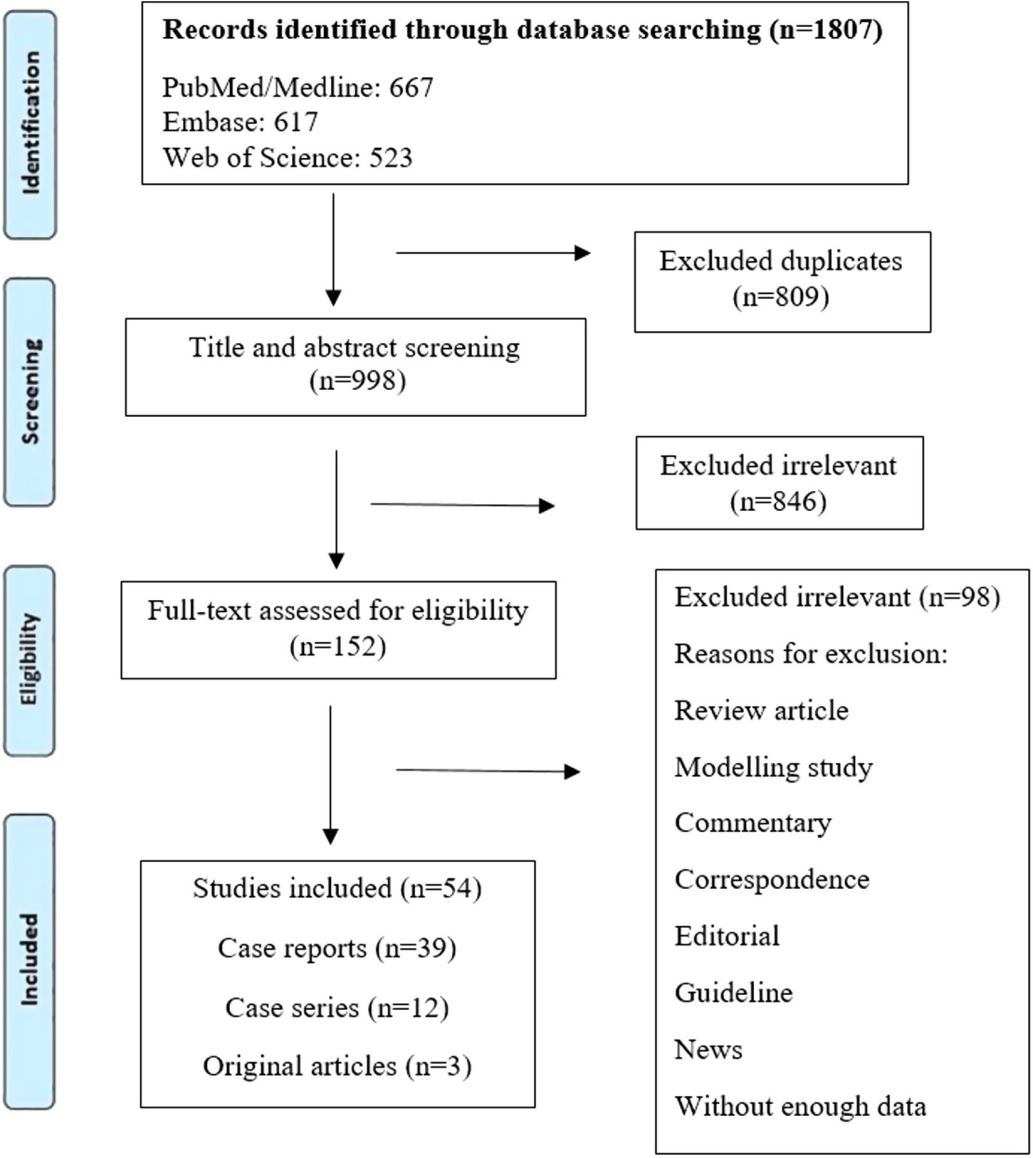
Flow diagram detailing review process and study selection

The search strategy yielded 1807 studies from three databases. Following the removal of duplicates, the title and abstract of 998 studies were examined. Among these studies, 152 were selected for full‐text assessment, and other 846 studies were eliminated due to irrelevancy. In all the selected studies, the relapse of coronavirus infection after a negative RT‐PCR test was reported. Among the 152 full‐text studies examined, only 54 studies were found to be eligible for data extraction (Figure 1). The included studies were original articles (5.5%, *N* = 3), case reports (72.2%, *N *= 39), and case series (22.2%, *N* = 12). Likewise, in the included studies, RT‐PCR tests were performed to detect both the first and the second infections. Thirteen studies were originated from Europe, 11 from the USA, 9 from China, and 6 from Brazil. These articles reported a total number of 207 patients who developed the second infection of coronavirus after a recovery, which was confirmed by a negative RT‐PCR test. Forty‐six studies reported the clinical features in the first infection; however, seven articles declared no symptoms. Only one study unrecorded the clinical features in the first infection. In addition, 42 investigations implied the medication and intervention.

In the second phase of infection, 43 articles reported the clinical manifestations, seven articles stated no sign, and four articles did not list any symptoms. Also, 37 studies reported specifically the treatment of the secondary infection. To compare the severity of symptoms between two phases of infection, sufficient information of both is required. Only 41 investigations presented features for both periods of infection. From these 41 studies, information of 63 cases was identified as qualified for the comparison of manifestations. Moreover, the approximate interval between negative and second positive RT‐PCR was obtained from 49 studies. The length of this interval reflects the characteristics of COVID‐19 relapse.

During diagnosis, evaluation, and treatment, attention to comorbidities is necessary. Among included studies, 44 articles specified comorbidities. Of these observations, 11 studies did not found any notable underlying conditions or disorders. In this survey, we examined the outcome of the COVID‐19 relapse, which was categorized into discharge or death. The outcome was reported for a total number of 199 patients from 49 studies. The detailed information of is summarized in Table 1.

### 3.2 Demographic and general information

Considering the studies included, we reviewed 207 patients presented with secondary infection of COVID‐19 after a period of recovery. A negative RT‐PCR confirmed the recovery from the first phase of disease. Among the included articles, there were only three observational studies that reported 132 cases. Of all 207 cases, 122 (58.9%) patients were female, and 85 (41.1%) patients were male. As shown in Table 2, children were less likely to have a recurrence of COVID‐19. However, the most patients were in the age group of 20–40 years. The studies reported a wide range of 2–240 days between two coronavirus infections. We classified this duration into three groups: *n* ≤ 30, 30 < *n* < 90, and *n* ≥ 90. Thirty‐eight (77.5%) studies reported *n* ≤ 30 or 30 < *n* < 90 for the recovery duration, and only 11 (22.5%) investigations implied more than 90 days (Table 2).

**TABLE 2:** Summary of the findings

	*n*/*N* (%)	No. of studies that mentioned
Sex		
Female	122/207 (58.94)	53
Male	85/207 (41.06)	
Age		
<20	1/207 (0.48)	1
20 ≤ *n* ≤ 40	157/207 (75.85)	21
40 < *n* < 90	29/207 (14.01)	16
≥90	20/207 (9.66)	15
Days between negative and second positive RT‐PCR		
≤30	23/49 (46.94)	49
30 < *n* < 90	15/49 (30.61)	
≥90	11/49 (22.45)	
Clinical manifestations in first infection		
Asthenia	34/51 (66.67)	15
Headache	46/69 (66.67)	22
Cough	52/95 (54.74)	34
Fever	55/104 (52.88)	43
Sore throat	33/66 (50)	19
Respiratory distress and signs	13/27 (48.15)	19
Myalgia	27/57 (47.37)	16
Diarrhea	27/68 (39.71)	14
Fatigue	14/37 (37.84)	20
Dyspnea	20/54 (37.04)	16
Sneeze	15/41 (36.59)	8
Chills	4/12 (33.33)	10
Hyporexia	13/42 (30.95)	9
Nausea	13/45 (28.89)	11
Abdominal pain	12/44 (27.27)	10
Malaise	3/11 (27.27)	10
Vomiting	11/42 (26.19)	9
Anosmia	15/60 (25)	14
Dysgeusia	11/44 (25)	8
Arthralgia	10/41 (24.39)	8
Dizziness	10/42 (23.81)	9
Tachypnea and respiratory alkalosis	3/14 (21.43)	10
Chest pain	4/19 (21.05)	11
Hypoxia	2/10 (20)	9
Skin lesions	8/41 (19.51)	8
Ageusia	3/19 (15.79)	10
Rhinorrhea	2/14 (14.29)	8
Drowsiness	1/9 (11.11)	8
Hypoxemia	1/9 (11.11)	8
Nasal congestion	1/9 (11.11)	8
Night sweat	1/9 (11.11)	8
Anorexia	1/14 (7.14)	8
Sputum	1/14 (7.14)	8
Mental confusion	2/41 (4.88)	8
Treatment in first infection		
Lopinavir/ritonavir	8/10 (80)	7
Oxygen support	9/13 (69.23)	11
Oseltamivir	4/6 (66.66)	6
Interferon	9/14 (64.29)	7
Chinese traditional medicine	10/16 (62.5)	6
Azithromycin	28/45 (62.22)	11
Antivirals	3/5 (60)	3
Immunoglobulin	3/5 (60)	5
Lopinavir	3/5 (60)	4
Vitamins	3/5 (60)	5
Zinc	3/5 (60)	5
Acetaminophen	2/4 (50)	4
Antibiotics	3/6 (50)	4
Arbidol	7/14 (50)	7
Ceftriaxone	5/10 (50)	5
Dexamethasone	2/4 (50)	4
Moxifloxacin	2/4 (50)	4
Tocilizumab	2/4 (50)	4
Methylprednisolone	3/7 (42.86)	5
Hydroxychloroquine	20/52 (38.46)	15
Albuterol	1/3 (33.33)	3
Amiodarone	1/3 (33.33)	3
Bisoprolol	1/3 (33.33)	3
Cefoperazone sodium	1/3 (33.33)	3
Cefuroxime	1/3 (33.33)	3
Chloroquine	1/3 (33.33)	3
Clindamycin	1/3 (33.33)	3
Daptomycin	1/3 (33.33)	3
Dipyrone	1/3 (33.33)	3
Enoxaparin	1/3 (33.33)	3
Furosemide	1/3 (33.33)	3
Home care	1/3 (33.33)	3
Montair	1/3 (33.33)	3
Naproxen	1/3 (33.33)	3
Paracetamol	1/3 (33.33)	3
Piperacillin/tazobactam	1/3 (33.33)	3
Prednisolone	2/6 (33.33)	4
Prednisone	1/3 (33.33)	3
Ranitidine	1/3 (33.33)	3
Remdesivir	2/6 (33.33)	4
Ribavirin	1/3 (33.33)	3
Sulbactam sodium	1/3 (33.33)	3
Thromboprophylaxis	1/3 (33.33)	3
Vancomycin	1/3 (33.33)	3
Ivermectin	10/37 (27.03)	4
Silybin	1/4 (25)	3
Antitussives	2/9 (22.22)	4
Corticosteroids and steroids	28/130 (21.54)	4
Anticoagulation	1/5 (20)	3
Levofloxacin	1/5 (20)	3
Ofloxacin	1/5 (20)	3
Rovamycine	1/5 (20)	3
Thymalfasin	1/6 (16.67)	3
Thymopentin	1/6 (16.67)	3
Heparin	5/35 (14.29)	3
Esomeprazole	1/8 (12.5)	3
Clinical manifestations in second infection		
Asthenia	34/54 (62.96)	13
Myalgia	42/67 (62.69)	23
Headache	44/72 (61.11)	21
Sore throat	31/59 (52.54)	18
Dyspnea	33/68 (48.53)	21
Sneeze	22/47 (46.81)	8
Diarrhea	25/58 (43.10)	15
Dysgeusia	22/53 (41.51)	11
Anosmia	24/62 (38.71)	14
Cough	60/171 (35.09)	28
Fatigue	10/31 (32.26)	15
Hyporexia	15/47 (31.91)	8
Arthralgia	15/48 (31.25)	9
Nausea	16/53 (30.19)	12
Vomiting	14/51 (27.45)	10
Abdominal pain	13/48 (27.08)	9
Dizziness	13/48 (27.08)	9
Malaise	5/19 (26.32)	12
Fever	46/186 (24.73)	37
Hypoxia	4/18 (22.22)	11
Tachypnea	4/18 (22.22)	11
Ageusia	5/25 (20)	12
Chills	5/28 (17.86)	12
Chest pain	4/25 (16)	11
Respiratory distress	18/127 (14.17)	23
Skin lesions and desquamation	6/48 (12.5)	9
Tachycardia	2/16 (12.5)	9
Mental confusion	6/49 (12.24)	9
Anorexia	1/15 (6.67)	8
Aphasia	1/15 (6.67)	8
Back pain	1/15 (6.67)	8
Coryzal symptoms	1/15 (6.67)	8
Gingival aphthous ulcers	1/15 (6.67)	8
Hypotension	1/15 (6.67)	8
Hyposmia	1/15 (6.67)	8
Hypoxemia	1/15 (6.67)	8
Insomnia	1/15 (6.67)	8
Lymphadenopathy	1/15 (6.67)	8
Nasal congestion	1/15 (6.67)	8
Rhinitis	1/15 (6.67)	8
Stranguria	1/15 (6.67)	8
Dehydration	1/17 (5.88)	8
Hypernatremia	1/17 (5.88)	8
Hyperthermia	1/17 (5.88)	8
Melena	1/17 (5.88)	8
Rhinorrhea	1/20 (5)	8
Sputum	1/20 (5)	8
Treatment in second infection		
Acyclovir	1/1 (100)	1
Ampicillin	1/1 (100)	1
Analgesics	1/1 (100)	1
Antipyretics	1/1 (100)	1
Acetaminophen	1/1 (100)	1
Arbidol	1/1 (100)	1
Budesonide	1/1 (100)	1
Caspofungin	1/1 (100)	1
Cefepime	1/1 (100)	1
Clarithromycin	2/2 (100)	2
Co‐amoxiclav	1/1 (100)	1
Colistin	1/1 (100)	1
CQ phosphate	1/1 (100)	1
Chinese traditional medicine	7/7 (100)	2
Dexamethasone	8/8 (100)	8
Enoxaparin	1/1 (100)	1
Favipiravir	2/2 (100)	2
Interferon	7/7 (100)	4
Linezolid	1/1 (100)	1
Lopinavir	1/1 (100)	1
Lopinavir/ritonavir	1/1 (100)	1
Meropenem	2/2 (100)	2
Montair	1/1 (100)	1
Moxifloxacin	1/1 (100)	1
Naproxen	1/1 (100)	1
Oseltamivir	2/2 (100)	2
Paracetamol	1/1 (100)	1
Polymixin	1/1 (100)	1
Ranitidine	1/1 (100)	1
Ribavirin	1/1 (100)	1
Piperacillin/tazobactam	2/2 (100)	2
Vancomycin	3/3 (100)	3
Vitamins	2/2 (100)	2
Zinc	2/2 (100)	2
Methylprednisolone	5/6 (83.33)	4
Corticosteroids and steroids	28/35 (80)	3
Remdesivir	8/10 (80)	8
Thymalfasin	3/4 (75)	1
Azithromycin	29/44 (65.91)	9
Ivermectin	23/35 (65.71)	2
Antibiotics	22/35 (62.86)	3
Ceftriaxone	4/7 (57.14)	4
Furosemide	2/4 (50)	2
Plasma	4/8 (50)	4
Oxygen support	21/54 (38.89)	17
Heparin	12/33 (36.36)	1
Aztreonam	1/3 (33.33)	1
Cotrimoxazole	1/3 (33.33)	1
Immunoglobulin	1/3 (33.33)	1
Levofloxacin	1/3 (33.33)	1
Tocilizumab	1/3 (33.33)	1
Hydroxychloroquine	13/44 (29.55)	7
Arbidol	1/4 (25)	1
Symptomatic care	1/6 (16.67)	1
Status		
Worse	29/63 (46.03)	41
Better	25/63 (39.68)	
ND	9/63 (14.29)	
Comorbidities		
Obesity	13/40 (32.5)	15
Kidney failure	8/26 (30.77)	17
Hypertension	19/63 (30.16)	24
Pneumonia	8/30 (26.67)	19
Heart failure	6/23 (26.09)	17
Arthritis	4/22 (18.18)	13
Hypothyroidism	3/17 (17.65)	14
Sleep apnea	3/17 (17.65)	14
Acute lymphoblastic leukemia	3/18 (16.67)	13
Diabetes	10/66 (15.15)	22
HIV	3/21 (14.29)	12
Liver failure	3/21 (14.29)	12
Sarcoidosis	3/21 (14.29)	12
Anxiety	2/16 (12.5)	13
Chemotherapy	2/16 (12.5)	13
Glucose/carbohydrate intolerance	2/16 (12.5)	13
Smoking	2/16 (12.5)	13
Under hemodialysis	2/16 (12.5)	13
Chronic obstructive pulmonary disease	2/18 (11.11)	12
Multiple myeloma	2/18 (11.11)	13
Alzheimer's disease and dementia	2/21 (9.52)	13
Dyslipidemia	2/21 (9.52)	13
Parkinson's disease	2/21 (9.52)	13
Asthma	5/53 (9.43)	16
Anemia	1/15 (6.67)	12
Atrial fibrillation	1/15 (6.67)	12
Bacterial meningitis	1/15 (6.67)	12
Beta‐lactam allergy	1/15 (6.67)	12
Cardiopulmonary arrest	1/15 (6.67)	12
Degenerative disk disease	1/15 (6.67)	12
End stage renal disease	1/15 (6.67)	12
Emphysema	1/15 (6.67)	12
Hepatitis B	1/15 (6.67)	12
Hypercalcemia	1/15 (6.67)	12
Hypercholesterolemia	1/15 (6.67)	12
Inflammatory syndrome	1/15 (6.67)	12
Lumbar fusion	1/15 (6.67)	12
Metastatic colorectal cancer	1/15 (6.67)	12
Nephropathy (diabetic)	1/15 (6.67)	12
Neuroblastoma	1/15 (6.67)	12
Neuropathy	1/15 (6.67)	12
Plasmacytoma	1/15 (6.67)	12
Respiratory failure	1/15 (6.67)	12
Urinary tract infection	1/15 (6.67)	12
Waldenstrom macroglobulinemia	1/15 (6.67)	12
Stroke	1/16 (6.25)	12
Digestive disease	1/17 (5.88)	12
Renal impairment	1/17 (5.88)	12
Allergic rhinitis	1/21 (4.76)	12
Depression	1/21 (4.76)	12
Outcome		
Discharge	190/199 (95.48)	49
Death	9/199 (4.52)	

Abbreviations: HIV, human immunodeficiency virus; *n*, number of patients with any variables; *N*, the total number of patients with COVID‐19; ND, no difference; No, number; RT‐PCR, reverse transcription‐polymerase chain reaction.

To compare the severity of the first infection with secondary, the reported features and symptoms were reviewed and extracted from the articles. Of 63 patients, 29 presented more severe manifestations, while 25 cases showed an ameliorated status, and 9 other cases indicated similar symptoms in both phases of the infection. Forty‐nine studies recorded the outcome of 199 patients, among whom 190 cases were discharged with an improved status, but 9 cases succumbed in hospital. Indeed, the survival rate is required to be taken into account when determining the potency of coronavirus reactivation.

### 3.3 Clinical manifestations

Some clinical signs were most frequent between the two infections, but prevalence was different. Moreover, the most prevalent clinical signs in the first infection were asthenia (66.6%), headache (66.6%), cough (54.7%), fever (52.8%), sore throat (50.0%), and respiratory distress (48.1%). However, in the second infection, asthenia (62.9%), myalgia (62.6%), headache (61.11%), sore throat (52.54%), and dyspnea (48.53%) were the common. Respiratory alkalosis (21.43%), drowsiness (11.11%), and night sweat (11.11%) occurred only in the first infection. The most frequent symptoms observed only in the second infection were as follows: asthenia (62.9%), myalgia (62.9%), and headache (61.1%). COVID‐19 recurrence was manifested with more mild signs in 39.6% of patients, while 46.0% presented with more severe signs, and other 14.3% did not show any prominent change between the two infections.

### 3.4 Treatment

Medications and treatments for the first COVID‐19 infection were reported in 40 studies. Among these treatments, hydroxychloroquine, azithromycin, and oxygen support were reported by 15, 11, and 11 articles, respectively. A total of 37 articles stated treatment for the second COVID‐19 infection. So that, oxygen support (17 studies), azithromycin (9 studies), dexamethasone (8 studies), and remdesivir (8 studies) were mostly reported. The most common treatment options used in first SARS‐CoV‐2 infection were lopinavir/ritonavir (80%), oxygen support (69.2%), and oseltamivir (66.6). However, for the treatment of second SARS‐CoV‐2 infection, mostly antibiotics (100%), dexamethasone (100%), and Remdesivir (80%) were used (Table 2).

### 3.5 Comorbidities

The evaluating comorbidities and underlying conditions can enlighten some aspects of COVID‐19. Based on the extracted data, a number of underlying diseases and conditions had a notable frequency. Obesity was highlighted as a condition in 32.5% of patients by 15 articles, whereas 22 studies stated diabetes with an overall prevalence of 15.15%. Hypertension and heart failure were reported to be 30.16% and 26.09% in 24 and 17 articles, respectively. Neurodegenerative disorders such as Alzheimer's (9.52%) and Parkinson's (9.52%) diseases were found as comorbidities (Table 2). However, evidence established an association between these types of comorbidities and COVID‐19; further investigations could clarify the detailed mechanisms of these relations.

## 4 DISCUSSION

To prevent reinfection or reactivation, four criteria can be considered for patients’ discharge. First, the patient should not have a fever for at least three days. Second, the patient's respiratory symptoms should considerably be ameliorated. Third, the radiological abnormalities shown in the CT scan and X‐ray images should substantially be improved. Four, as per WHO recommendation, patients should have two consecutive negative RT‐PCR results with a 24‐h interval.^14^

Improved or discharged patients are connected to the members of the community, and they, therefore, are presented as a latent source of infection.^14^ In this study, we assessed the prevalence and frequency of recurrence or reinfection in patients with COVID‐19 and performed investigations from various aspects, including factors related to host, virus, and environment. The emergence of new virus mutations is the main hypothesis on mechanisms of the COVID‐19 reinfection. The new variants of the SARS‐CoV‐2 can bind to human cells, and the produced antibodies in the first infection could not efficiently opsonize them. Actually, these variants can lead to evading the immune response.^15^

Several factors influencing reinfection, including the initial load of the virus and the type of genome, are virus‐dependent.^16^ The average duration of SARS‐CoV‐2 shedding is 20 days, which in some cases is 37 days.^17^ A survey has suggested an average viral shedding of 53 days, with a maximum of 83 days. Patients in whom clinical symptoms had started earlier tented to have a longer duration of viral shedding and more severe disease.^18^

The study by Elrashdy et al. found that the average time period between the previous discharge and the next positive test was 4–17 days.^14^ In the present study, the highest incidence of reinfection was related to a period of less than 30 days (46.94%). In the periods of 30–90 days, the incidence of reinfection was 30.61%, and the lowest incidence (22.45%) was observed in the period of more than 90 days. Given the studies reviewed above, there is a discrepancy between the duration of subsequent coronavirus infection and the antibody‐induced immunity. Therefore, there is certainly other factors, such as the level of the individual's immune system or the accuracy of the tests, that affect this time period. Perhaps, the reason for the recurrence of the disease 7–14 days after discharge from the hospital is that the virus is still hidden in exosomes or extracellular vesicles and resumes activity after a period of "silences".^14^

In this study, RT‐PCR was a necessary inclusion criterion. Thus, patients with only a serologic diagnosis test, without a nasopharyngeal swab RT‐PCR were excluded. RT‐PCR is the gold standard for diagnosing SARS‐CoV‐2; however, this test has low sensitivity due to test error or insufficient sample size.^19^ The accuracy of RT‐PCR is 97%,^20^ and the occurrence of false negatives in PCR of SARS‐CoV‐2 has been reported to be 30%,^21^ which in some cases increases due to sampling error.^20^ One of the reasons for the error in RT‐PCR is the prolonged conversion of nucleic acid, which causes recurrence or "turn positive".^22^ In the early stages of infection, the SARS‐CoV‐2 is readily detected in the upper respiratory tract. As the disease progresses, the virus appears in the lower respiratory tract and other organs such as the intestines and blood.^23^ Therefore, it is impossible to identify SARS‐CoV‐2 in the throat, and some patients may have positive CT scan, despite the negative RT‐PCR.^24^

Incorrect sampling is another reason for recurrence in improved individuals,^14^ although it is unlikely to happen due to the use of devices such as gloves, masks, and caps.^25^

As the laboratory detection of virus nucleic acid can have false‐negative results, serological tests for specific IgG and IgM can be alternated.^19^ Therefore, PCR alone is not adequate for discharging patients from hospital. Supplementary tests such as serological ones, together with the criteria recommended by the WHO and other specific health organizations, are needed to be performed in every country. There is a period of time between the apparent recovery in the clinic and the complete recovery from the SARS‐CoV‐2. Viral carriers with low symptoms pose a greater challenge to epidemic management and control.^26^ Conducting two negative PCR at 24‐hour intervals is insufficient for detecting the virus; thus, repeating the test for a longer period of 48 h is recommended. In addition, immunological tests such as d‐dimer and absolute lymphocyte counts and even antibody testing should be performed. RT‐PCR results are negative on average 2.73 days after hospitalization.^26^ Wolfel et al. in their study evaluated the hospitalized patients with COVID‐19. They demonstrated that after eight days of infection, the live virus is undetectable.^27^

Gender, old age, and the type of disease are host‐dependent factors influencing the occurrence of reinfection and require immune system suppression.^16^ In the present study, women became more infected than men (58.94% vs. 41.06). Children were less likely to have COVID‐19 relapse. However, the most patients were in the age group of 20–40 years. In addition, obesity (32.5%), kidney failure (30.7%), and hypertension (30.1%) were the most frequent underlying comorbidities observed among COVID‐19 relapse patients. Although studies have shown that underlying conditions cause the severity of COVID‐19 disease, but how each of these factors contribute to reinfection should be examined by designing new studies determining these effects separately or in combination.^28^, ^29^

The clinical features of patients with reinfection are similar to those of primary infection. The presence of asymptomatic patients among reactivated patients caused the recurrence of the asymptomatic contamination or infection with few symptoms.^14^, ^16^

In an earlier study, rhesus macaques became reinfected after recovery, without showing any symptoms. This finding highlights the need for strict protection from SARS‐CoV‐2 and its control, to hider the development of this severe disease.^30^ The second time of infection severity is varied; some cases show mild, and some others indicate more severe symptoms.^10^ In a former study, 46.03% of patients had a worse condition, and 39.68% had a better condition in the second than the first infection. One of the reasons for the deterioration condition of patients in the second infection, compared with first one, is the occurrence of an antibody‐dependent enhancement (ADE) that increases the infectivity of virus in the secondary infection.^31^ However, a patient with strong immunity and more immune memory cells and T‐cell mediation could decrease the severity of the second infection.^10^

Normally, people with a primary infection with mild symptoms and those with a suppressed immune system are more likely to get COVID‐19 for the second time because they do not produce an adequate immune response. It has also been demonstrated that 95.48% of the patients reinfected with the SARS‐CoV‐2 were discharged from hospital and 4.52% were died. The rate of death would have possibly been reduced if patients had received more care during their first‐time hospitalization.^10^

SARS‐CoV‐2 reactivation may occur when using any antiviral drug.^16^ In this survey, the most recurrence of the disease occurred after taking lopinavir/ritonavir, oseltamivir, interferon, and Chinese traditional medicine. These drugs may not have been able to fully eradicate viruses from the body, and some of them may remain in the body, causing reinfection. However, further investigation can evaluate the effectiveness of different drugs in complete eradication of the virus to eliminate the possibility of reinfection.

In a study performed by Okhuese, the proportion of infected population will continue to grow in the world if unvaccinated. At the same time, the rate of recovery will continue slowly. In other words, in this situation, the mortality rate can be determined based on the ratio of infection to recovery rate. The rate of reinfection with clinical clearance of the virus from the improved population decreases to zero over time.^32^ Contrary to the results achieved in Okhuese's study,^33^ despite the high prevalence of SARS‐CoV‐2, the rate of reinfection is still high. Therefore, more experimental and laboratory studies are needed to determine the cause of reinfection and its frequency. Of note, reinfection differs from reactivation. Reinfection is caused by different variants of SARS‐CoV‐2 virus, but reinfection occurs with the same strain. The only way to discriminate the reinfection and reactivation is by sequencing and molecular techniques.^34^ Regrettably, the first two actions happen only in 5%–10%.^10^ Designing studies to sequence the virus genome in the first and second infections is highly recommended. In this way, the cause of COVID‐19 recurrence is clarified, and its prevalence in the community is determined.

A number of limitations can be considered in this study. The first is the small number of original articles and short communication. The second is related to case series and case reports studies, which lack sufficient and accurate information on patients and are often reported descriptively. Therefore, accurate meta‐analysis calculations were impossible in this study.

## 5 CONCLUSION

The present study represents a large number of COVID‐19 reactivation over different countries. Overall, the recurrence of COVID‐19 in recovered patients may arises from various factors, including a false negative or positive in PCR, differences in tests, incorrect diagnosis by physicians to discharge a patient with COVID‐19, illness for reasons other than COVID‐19, the presence of various strains of SARS‐CoV‐2, and dysfunction of immune systems. Our results highlighted the potency of COVID‐19 recurrence as an outstanding issue. This feature needs to be regarded in the management of COVID‐19. The first and second COVID‐19 are the same in terms of clinical manifestations, but they are not distinguishable. So far, no acceptable marker has been found to predict the risk of reinfection. In addition, there is no validated test of whether a particular drug or treatment is associated with reinfection or reactivation. A careful follow‐up of discharged patients and accuracy in their discharge and removal from quarantine is of paramount importance to inhibit reinfection. Given the data discussed in this work, the first coronavirus infection can lead to the recurrence of COVID‐19. Regarding COVID‐19 infection, there are two hypothesis: (a) COVID‐19 infection reactivates following a period of dormancy, and (b) COVID‐19 increases the susceptibility to the second coronavirus invasion. Future experimental and clinical researches could examine these hypotheses and finally provide a clear view of COVID‐19 relapse.

## CONFLICT OF INTEREST

The authors declare that they have no competing interests.

## AUTHORS’ CONTRIBUTION

Maryam Koupaei, Mohamad Hosein Mohamadi, Ilya Yashmi, Amir Hossein Shahabi, Amir Hosein Shabani, Mohsen Heidary, and Saeed Khoshnood contributed in revising and final approval of the version to be published. All authors agreed and confirmed the study for publication.

## INFORMED CONSENT

Not applicable.

## CONSENT FOR PUBLICATION

Not applicable.

## Data Availability

All the data in this review are included in the study.
